# Bailout transcatheter edge-to-edge mitral valve repair for acute mitral regurgitation attributable to systolic anterior motion during transcatheter aortic valve implantation: a case report

**DOI:** 10.1093/ehjcr/ytag452

**Published:** 2026-06-22

**Authors:** Yuki Numata, Suguru Hirose, Riichi Nishikawa, Shigeru Toyoda, Shinsuke Hamaguchi

**Affiliations:** Department of Anesthesiology, Dokkyo Medical University Hospital, 880 Kitakobayashi, Mibu, Shimotsuga, Tochigi 321-0293, Japan; Department of Cardiovascular Medicine, Dokkyo Medical University Hospital, 880 Kitakobayashi, Mibu, Shimotsuga, Tochigi 321-0293, Japan; Department of Cardiovascular Medicine, Dokkyo Medical University Hospital, 880 Kitakobayashi, Mibu, Shimotsuga, Tochigi 321-0293, Japan; Department of Cardiovascular Medicine, Dokkyo Medical University Hospital, 880 Kitakobayashi, Mibu, Shimotsuga, Tochigi 321-0293, Japan; Department of Anesthesiology, Dokkyo Medical University Hospital, 880 Kitakobayashi, Mibu, Shimotsuga, Tochigi 321-0293, Japan

**Keywords:** Aortic stenosis, Mitral valve regurgitation, Systolic anterior motion, Transcatheter aortic valve implantation, Transcatheter edge-to-edge mitral valve repair, Case report

## Abstract

**Background:**

Systolic anterior motion (SAM) of the mitral valve is a rare but life-threatening complication of transcatheter aortic valve implantation (TAVI) that can cause acute severe mitral regurgitation (MR) and/or left ventricular outflow tract (LVOT) obstruction, leading to rapid haemodynamic deterioration. Evidence supporting the use of transcatheter edge-to-edge mitral valve repair (M-TEER) for SAM-related MR during TAVI, particularly in the absence of significant LVOT obstruction, is limited.

**Case summary:**

A 78-year-old woman with severe symptomatic aortic stenosis and multiple comorbidities underwent transfemoral TAVI. Immediately after predilatation, she experienced haemodynamic collapse and required percutaneous cardiopulmonary support. Transoesophageal echocardiography revealed SAM of the anterior mitral leaflet with severe MR without apparent LVOT obstruction. Although a transcatheter aortic valve was subsequently implanted, severe MR with haemodynamic instability persisted. As medical management did not stabilize haemodynamics, bailout M-TEER was performed 3 days after TAVI. Thereafter, MR improved from severe to mild and haemodynamic stabilization rapidly occurred. The patient was discharged with New York Heart Association class I and remained asymptomatic. Sustained MR improvement was observed during 6-month follow-up.

**Discussion:**

In this case, SAM-related MR without apparent LVOT obstruction developed immediately after predilatation in a patient with a sigmoid septum, and off-label M-TEER achieved haemodynamic stabilization. Although evidence supporting M-TEER for SAM-related MR during TAVI is limited to case reports and small case series, this case suggests that M-TEER may be considered a bailout option for selected patients when MR is the primary cause of haemodynamic collapse, even without apparent LVOT obstruction.

Learning points

- SAM-related MR can be a primary cause of acute haemodynamic deterioration during TAVI, even when the LVOT pressure gradient is not pronounced.
- Preoperative assessment of morphological risk factors for SAM, such as sigmoid septum anatomy, should be included in routine TAVI planning.
- For haemodynamic deterioration predominantly due to SAM-related MR, M-TEER may be an effective treatment option. However, as this is an off-label therapy, careful risk management is essential.

## Introduction

Transcatheter aortic valve implantation (TAVI) is a standard treatment for severe aortic stenosis (AS).^1–3^ However, a potential periprocedural complication is systolic anterior motion (SAM) of the mitral valve. Although SAM is rare and affects only approximately 1.6% of patients,^4^ it represents a serious complication that can cause severe mitral regurgitation (MR) and left ventricular outflow tract (LVOT) obstruction, potentially leading to acute haemodynamic collapse.

The pathophysiology of SAM cannot be explained by the classical Venturi effect alone. Recent studies have suggested a multifactorial mechanism involving fluid dynamic forces acting on an anatomically predisposed left ventricular–mitral valve complex. When SAM-related MR remains refractory to medical and haemodynamic management, surgical interventions may be required. Transcatheter edge-to-edge mitral valve repair (M-TEER) reduces MR by approximating the mitral leaflets and enhancing coaptation. Because M-TEER for SAM-related MR during TAVI is off-label, evidence is limited to case reports and small case series. Nevertheless, in cases of mild or absent LVOT obstruction and MR as the predominant cause of haemodynamic instability, M-TEER may be a viable rescue option. We report a case of symptomatic SAM-related MR during TAVI without a significant LVOT gradient in which haemodynamic stabilization was achieved through rescue M-TEER.

## Summary figure: timeline

**Table:** 

Time point	Events
Baseline	The patient presented with symptomatic severe aortic stenosis and heart failure. Transthoracic echocardiography revealed a sigmoid septum, no evidence of left ventricular outflow tract obstruction, and mild mitral regurgitation.
Day 0 (TAVI)	Transfemoral TAVI was performed on day 0. After balloon pre-dilatation, profound hypotension occurred, requiring cardiopulmonary resuscitation and percutaneous cardiopulmonary support. Transoesophageal echocardiography demonstrated systolic anterior motion of the anterior mitral leaflet with severe mitral regurgitation. A 25-mm Navitor valve was implanted; however, the severe mitral regurgitation and haemodynamic instability persisted.
Day 0–2	Conventional medical therapy and mechanical circulatory support failed to resolve the severe mitral regurgitation over Days 0−2, and rescue M-TEER was planned.
Day 3 (M-TEER)	M-TEER was performed using a MitraClip NT implanted at the central A2–P2 segment. Mitral regurgitation improved from severe to mild, and haemodynamics stabilized rapidly.
Day 40	The transcatheter heart valve function remained normal with mild residual mitral regurgitation, and the patient was discharged with a NYHA functional class I status.
6-month follow-up	The patient remained asymptomatic, with mild residual mitral regurgitation and no readmissions for heart failure.

## Case presentation

**Figure 1:**
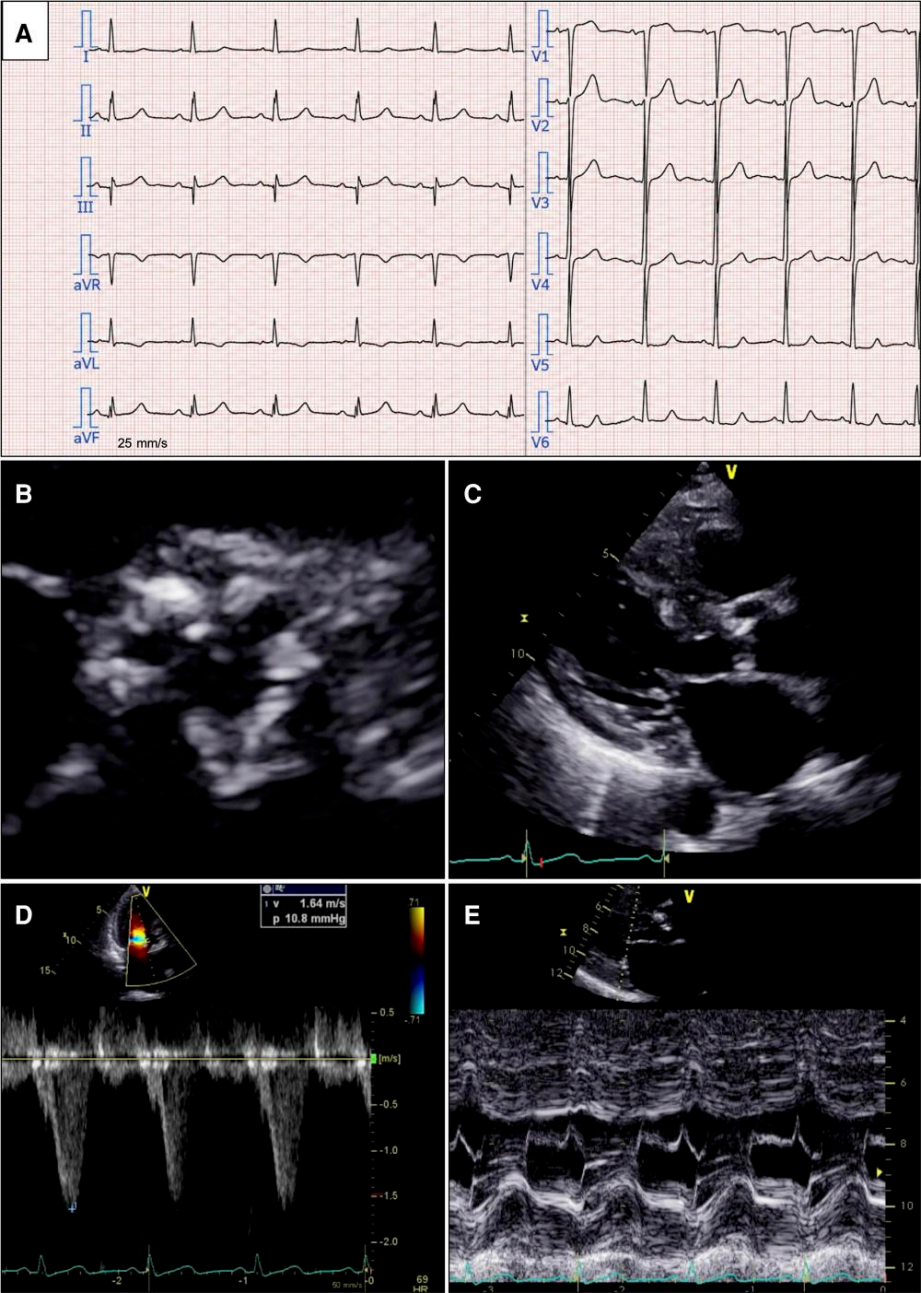
Preprocedural electrocardiography and transthoracic echocardiography. (*A*) Electrocardiogram. (*B*) Parasternal short-axis view showing a severely calcified aortic valve. (*C*) The interventricular septum has a sigmoid configuration and a basal septal bulge without evidence of asymmetric septal hypertrophy. (*D*) Pulsed-wave Doppler image of the left ventricular outflow tract (LVOT) showing no significant obstruction. (*E*) M-mode image reveals no evidence of overt systolic anterior motion (SAM).

**Figure 2:**
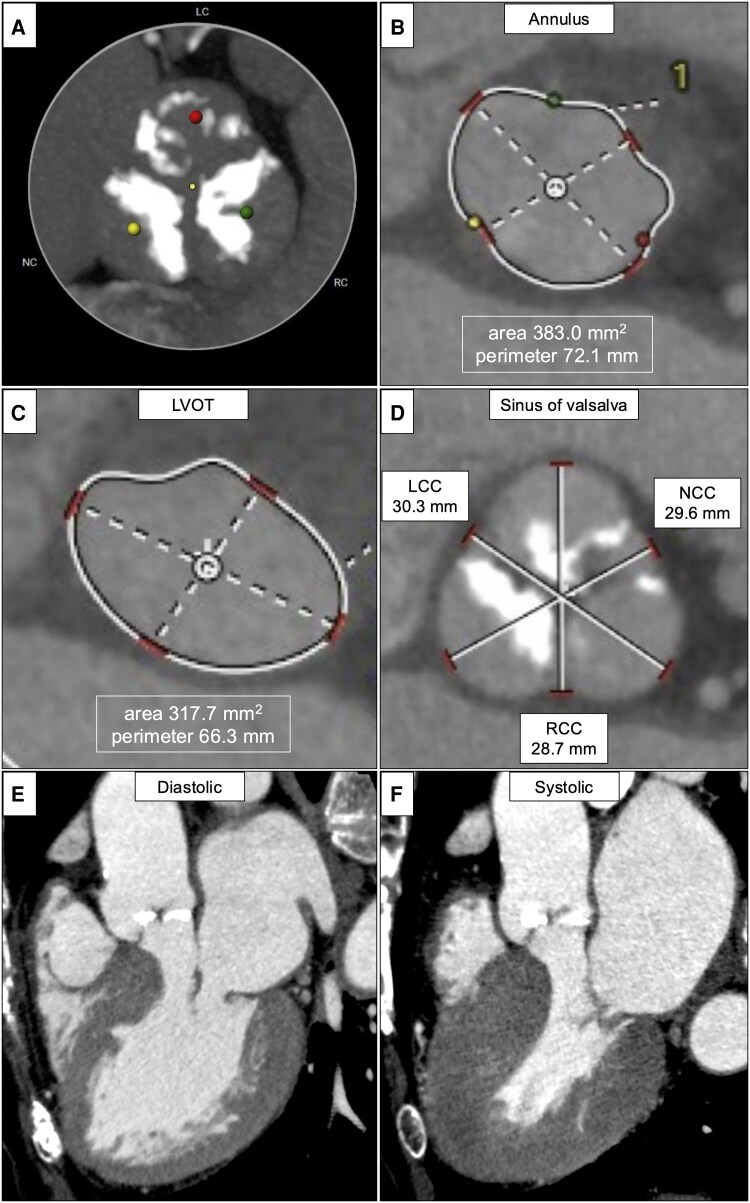
Preprocedural computed tomography (CT) findings of the periaortic anatomy and left ventricle. (*A*) Tricuspid aortic valve with severe calcification. (*B–D*) CT images showing anatomical structures surrounding the aortic valve and relevant measurements. (*E*, *F*) Diastolic and systolic long-axis CT views showing a bulging basal interventricular septum. The anterior mitral leaflet does not exhibit systolic anterior motion and asymmetric septal hypertrophy is not evident. Abbreviations: LCC, left coronary cusp; LVOT, left ventricular outflow tract; NCC, noncoronary cusp; RCC, right coronary cusp.

**Figure 3:**
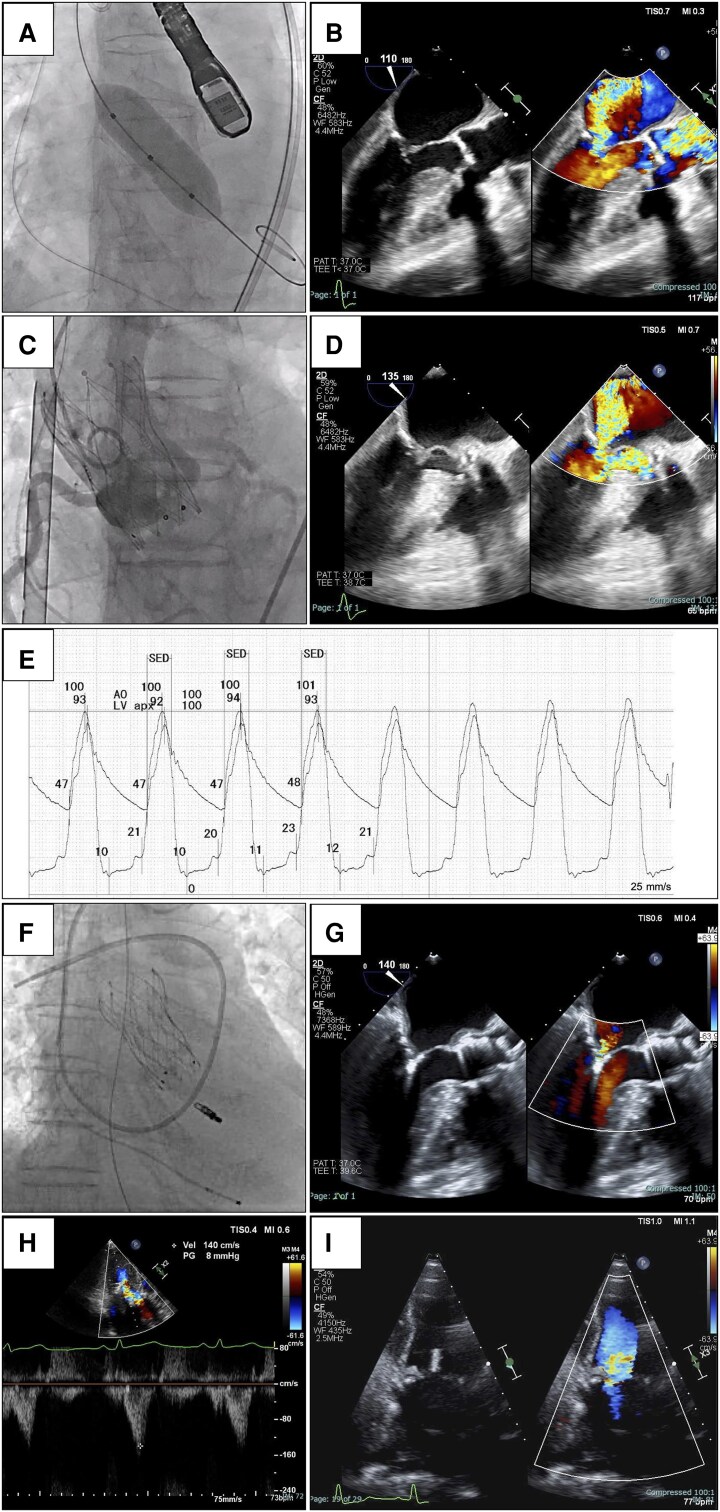
Angiography and transoesophageal echocardiographic findings during the periprocedural course. (*A*) Fluoroscopic image during balloon predilatation using an 18-mm balloon. (*B*) Transoesophageal echocardiography image immediately after predilatation showing mitral leaflet malcoaptation and severe mitral regurgitation. (*C*) Aortography after implantation of a 25-mm Navitor valve. (*D*) Severe mitral regurgitation (MR) persisting after valve deployment. (*E*) Simultaneous pressure measurement before mitral transcatheter edge-to-edge repair (M-TEER) shows no significant left ventricular outflow tract (LVOT) pressure gradient. (*F*) After deployment of MitraClip NT at the central A2–P2 segment. (*G*) After M-TEER, the MR improved from severe to mild. (*H*) LVOT flow acceleration is not observed after M-TEER. (*I*) Follow-up echocardiography image showing mild residual mitral regurgitation. Abbreviations: LVOT, left ventricular outflow tract; MR, mitral regurgitation; M-TEER, mitral transcatheter edge-to-edge repair; TAVI, transcatheter aortic valve implantation.

A 78-year-old woman with severe symptomatic AS and heart failure was referred to our hospital for intervention. Her comorbidities included rheumatoid arthritis, interstitial pneumonia, and prior subarachnoid haemorrhage. She was receiving long-term oral prednisolone (5 mg daily). Electrocardiography showed left ventricular hypertrophy, and transthoracic echocardiography (TTE) revealed severe AS (valve area, 1.0 cm^2^; aortic velocity, 4.7 m/s; mean pressure gradient, 48 mmHg) with a left ventricular ejection fraction of 69% and mild MR (*Figure 1A, B*). The interventricular septal and posterior wall thicknesses were 12 and 11 mm, respectively, with a sigmoid septum (*Figure 1C*, Supplementary Movie 1). No evidence of LVOT obstruction (peak velocity, 1.6 m/s) and no SAM of the anterior mitral leaflet (*Figure 1D, E*) were observed. Computed tomography revealed a severely calcified aortic valve and narrow LVOT with basal septal bulging (*Figure 2*). The surgical risk was moderate (Society of Thoracic Surgeons predicted mortality score, 7.7%); therefore, TAVI was performed under general anaesthesia via a transfemoral approach. Immediately after predilatation with an 18-mm Z-Med balloon (NuMED, Hopkinton, NY, USA), she developed profound hypotension, requiring cardiopulmonary resuscitation and veno-arterial extracorporeal membrane oxygenation (VA-ECMO) (*Figure 3A*). Angiography excluded annular rupture or coronary obstruction, and no lethal arrhythmias were observed. Transoesophageal echocardiography (TEE) ruled out acute severe aortic regurgitation and pericardial effusion but revealed SAM of the anterior mitral leaflet with severe MR with blunted pulmonary venous systolic flow and partial systolic flow reversal (*Figure 3B*). A 25-mm Navitor (Abbott, Santa Clara, CA, USA) was successfully deployed under VA-ECMO support (*Figure 3C*). After valve deployment, the patient was initially weaned from VA-ECMO; however, severe MR and haemodynamic instability persisted (*Figure 3D*, **Supplementary Movie 2**). Noradrenaline infusion (0.05–0.2 μg/kg/min) and volume loading were used to treat her hypotension. Because of suspected hypertrophic obstructive cardiomyopathy-like haemodynamics, temporary pacing and cibenzoline (168 mg/day) were attempted; however, the MR did not improve and cibenzoline was discontinued. Intra-aortic balloon pump (IABP) was also initiated; however, the MR worsened, possibly due to afterload reduction. Additionally, low-dose continuous furosemide infusion was attempted for congestion related to volume overload, but was discontinued after abrupt hypotension occurred during diuresis. An invasive pressure measurement showed a mild LVOT gradient (*Figure 3E*) without significant LVOT obstruction. Collectively, these findings suggested that SAM-related MR was the predominant driver of haemodynamic instability; therefore, early MR reduction was deemed necessary.

Because surgical intervention was considered excessively high-risk (recalculated STS score, 8.9％), the Heart Team decided to proceed with bailout M-TEER despite its off-label status. M-TEER was performed under VA-ECMO support 3 days after TAVI. Because the mitral valve area was relatively small (3.61 cm^2^) and iatrogenic mitral stenosis was a concern, a single-clip strategy was adopted. A MitraClip NT (Abbott, Santa Clara, CA, USA) was implanted at the central A2–P2 scallops (*Figure 3F, G*). The final mean transmitral gradient was 5 mmHg. The MR improved from severe to mild (see Supplementary Movie 3**)**, prompting haemodynamic stabilization. Pre-discharge TTE revealed satisfactory bioprosthetic valve function (effective orifice area, 2.7 cm^2^; mean gradient, 12 mmHg; left ventricular ejection fraction, 64%), mild residual MR, and no LVOT flow acceleration *(Figure 3H, I*). The patient was discharged on postoperative day 40 (New York Heart Association functional class I). At 6 months, the MR remained mild and readmission was not required.

## Discussion

In our case, severe SAM-related MR emerged immediately after predilatation and precipitated haemodynamic collapse. Although a significant LVOT pressure gradient was not observed during assessment, SAM-related MR was considered the main cause of haemodynamic instability. Because the pressure gradient assessment was performed under complex acute phase conditions, including mechanical circulatory support and pharmacological management, transient dynamic LVOT obstruction could not be completely excluded. Therefore, this case should be interpreted within the continuum of SAM physiology influenced by abrupt loading changes after TAVI. Previously reported cases of SAM-related MR after TAVI treated with M-TEER mainly involved overt LVOT obstruction or hypertrophic obstructive cardiomyopathy. Detailed reports of SAM-related MR after TAVI are limited.^5–7^

Mechanistically, SAM has traditionally been attributed to Venturi-related suction; however, accumulating evidence supports a predominant role for drag forces. Ro *et al*. described ‘vortical SAM,’ which is triggered by early systolic intraventricular vortex flow; however, Guigui *et al*. described ‘ejection SAM,’ whereby the ejection jet pushes the anterior leaflet towards the septum during systole, suggesting a two-step process.^8,9^ In such drag-dominant models, severe MR attributable to malcoaptation may occur even without a prominent LVOT gradient at the time of evaluation.

**Figure 4:**
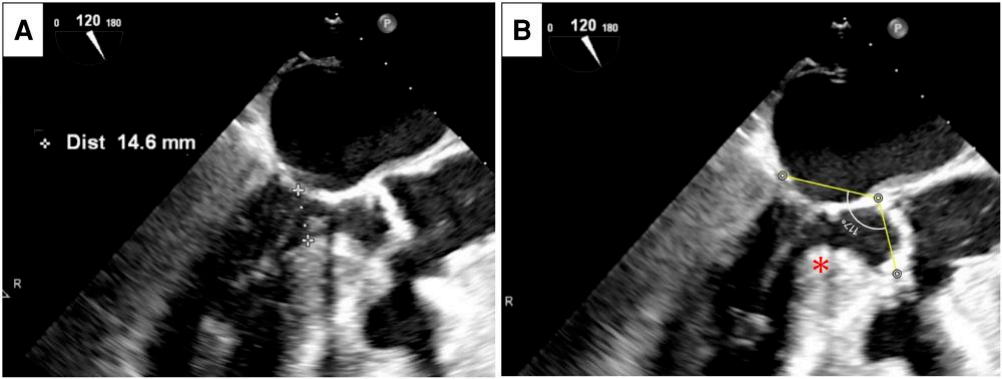
Morphologic risk factors for systolic anterior motion in our patient. (*A*) Transoesophageal echocardiography showing a short distance (Dist) between the coaptation point and septum (14.6 mm). (*B*) The aortomitral angle measured in the same view is narrow (117°). The basal interventricular septum has a sigmoid configuration (*).

In this patient, a short distance between the coaptation point and septum (14.6 mm), narrow aortomitral angle (117°), and sigmoid septum were present (*Figure 4A, B*), consistent with the morphological features associated with SAM.^10,11^ These preprocedural anatomical factors may provide a foundation for SAM-related MR or LVOT obstruction triggered by abrupt changes in loading conditions after TAVI. Therefore, a preprocedural assessment of the LVOT–mitral valve complex morphology may identify high-risk patients and guide periprocedural management strategies (*Table 1*).^9,10,12–14^

**Table 1:** Preprocedural assessment of systolic anterior motion-related mitral regurgitation after transcatheter aortic valve implantation

Domain	Features assessed	Findings suggestive of susceptibility	Findings of this case
LV/LVOT morphology	LV cavity size	Small or underfilled LV cavity	None
	Septal morphology	Sigmoid septum or basal septal bulging	Present
	LVOT geometry	Narrow LVOT relative to the aortic annulus	Present
Mitral valve–LVOT complex	Mitral leaflet morphology	Elongated/redundant mitral leaflet or leaflet disproportion	None
	Coaptation–septum relationship	Short C-sept distance	Present, 14.6 mm
	Aortomitral relationship	Acute aortomitral angle	Present, 117°
Haemodynamic features	LV contractility	Preserved or hyperdynamic LV function	Present, LVEF 69%

C-sept distance, distance between the coaptation point and septum; LV, left ventricular; LVEF, left ventricular ejection fraction; LVOT, left ventricular outflow tract; SAM, systolic anterior motion; TAVI, transcatheter aortic valve implantation.

The features listed in this table were selected based on previous reports of SAM after TAVI, dynamic LVOT obstruction, and SAM after mitral valve surgery.

When managing SAM-related haemodynamic compromise, excessive preload/afterload reduction must be avoided. In our patient, IABP aggravated MR, consistent with the paradoxical worsening of SAM-related MR with afterload reduction.^9,12^ M-TEER rapidly reduced MR by improving central coaptation and limiting anterior leaflet excursion, thereby stabilizing haemodynamics. Because of the relatively small mitral valve area, MR reduction was balanced with the risk of iatrogenic mitral stenosis. After single-clip implantation, the final mean transmitral gradient was 5 mmHg, which is less than the commonly used cautionary threshold (>5 mmHg). Therefore, it was considered acceptable in a bailout setting.

Although off-label and supported by limited evidence, M-TEER may be a bailout option when MR is the predominant cause of haemodynamic instability without a significant LVOT gradient. Preprocedural LVOT–mitral valve complex assessments may identify high-risk patients and guide periprocedural management. However, follow-up was limited to 6 months, thus precluding long-term MR durability and haemodynamics assessments; further cases are needed to establish optimal assessment and treatment strategies for SAM-related MR after TAVI.

## Supplementary Material

ytag452_Supplementary_Data

## Data Availability

The data underlying this article are available from the corresponding author upon reasonable request.

## Lead author biography

Yuki Numata, MD, PhD, is a Lecturer in the Department of Anesthesiology at Dokkyo Medical University Hospital, Tochigi, Japan. He is board-certified in cardiovascular anaesthesia and focuses on perioperative anaesthetic management for cardiovascular surgery and transcatheter structural heart interventions. He is committed to improving the quality of perioperative care for patients undergoing cardiovascular procedures.

## Notes

**Consent:** The authors confirm that written consent for submission and publication of this case report, including images and associated text, has been obtained from the patient in line with COPE guidance. Written informed consent was obtained from the patient for the publication of this case and accompanying images.
